# Predicting response to infliximab and interferon-α in Behçet’s syndrome: An exploratory analysis from the BIO-BEHÇET’S randomized controlled trial

**DOI:** 10.1515/rir-2025-0010

**Published:** 2025-07-01

**Authors:** Robert J. Moots, Farida Fortune, Richard Jackson, Tony Thornburn, Ann W. Morgan, Daniel F. Carr, Philip I. Murray, Christian Ludwig, Graham Wallace, Deva Situnayake

**Affiliations:** Faculty of Health, Social Care and Medicine, Edge Hill University, Ormskirk, UK; Department of Academic Rheumatology, Liverpool University Hospitals NHS Foundation Trust, Aintree University Hospital, Longmoor Lane, Liverpool, L9 7AL, UK; Queen Mary University of London, UK; Barts Health NHS Trust, The London Hospital, London UK; Liverpool Clinical Trials Centre, University of Liverpool, Liverpool UK; Behçet’s UK, Kemp House, 152–160 City Road, London, UK; Leeds Institute of Cardiovascular and Metabolic Medicine, University of Leeds, UK and NIHR Leeds Biomedical Research Centre, Leeds Teaching Hospitals NHS Trust, Leeds UK; Institute of Systems, Molecular and Integrated Biology, University of Liverpool, Liverpool UK; Department of Inflammation and Ageing, University of Birmingham, Birmingham UK; Department of Rheumatology, Sandwell and West Birmingham Hospitals, Birmingham UK

**Keywords:** Behçet’s, infliximab, interferon, metabolomic, IFNL3

## Abstract

**Background and Objectives:**

Biologic therapy has been used for Behçet’s Syndrome after first-line immunomodulation, but in the absence of high-quality evidence or predictive biomarkers. BIO-BEHÇET’S was a randomized controlled clinical trial to compare the two most widely used biologics for Behçet’s Syndrome at that time, infliximab versus interferon-α2a, and identify potential biomarkers for response.

**Methods:**

A total of 79 patients with active Behçet’s Syndrome were randomized to either infliximab (REMICADE) or interferon-α2a (ROFERON) according to the UK national treatment pathway, and follow-up with symptom-directed examination undertaken at Weeks 12 and 24. The head-to-head trial included an exploratory analysis on the potential role of single nucleotide polymorphisms (SNPs) and urinary metabolomic to act as biomarkers for drug response. Genotypic analysis was performed to determine whether four SNPs in *IFNL3* and *IFNL4* – selected based on known effects – impacted primary and secondary outcomes. For metabolomic analyses, urine samples were analyzed by nuclear magnetic resonance spectroscopy and principal component analysis.

**Results:**

Genetic data suggested potential association between outcomes and carriage of rs4803221 or rs7248668 variants in the *IFNL3* (IL-28B) gene locus for interferon-α2a patients; however, with the relatively small sample, statistical significance was lost when corrected for multiple testing. Metabolomic analysis identified potential markers of metabolic response to infliximab.

**Conclusion:**

BIO-BEHÇET’S suggests there is potential for a novel metabolomic biomarker that can identify response to infliximab in patients with Behçet’s Syndrome. Further work will characterize the appropriate metabolite (s) from existing samples to inform future prospective trials to study this in more detail clinically.

## Background

Behçet’s Syndrome is classified as a systemic variable vessel vasculitis,^[1]^ but understanding of the underlying pathophysiology is limited – although it is known that the tumor necrosis factor (TNF) pathway plays a pivotal role,^[2]^ and both TNF inhibitors and interferon-α biologics show good outcomes in non-responders to first-line corticosteroids and immunomodulators.^[3, 4, 5]^ Behçet’s Syndrome is thought to be a polygenic autoinflammatory disorder driven through epistatic interaction between *HLA-B51* and *ERAP1* gene variants, which influence peptide processing and presentation.^[6]^ Other genetic risk factors vary by population.^[6, 7, 8]^ As in other complex chronic diseases there is a need to better understand the phenotype of Behçet’s Syndrome patients in clinical trials, and to explore potential biomarkers that may help target therapies and minimize adverse events – since no such biomarkers are currently available. To address this, an exploratory analysis of two genetic and metabolomic biomarkers was built into the BIO-BEHÇET’S study – a pragmatic, standard-of-care, randomized, two-arm, parallel trial comparing biologics after first-line failure. The clinical findings have been published separately.^[9]^

Three genome-wide association studies in patients with hepatitis C virus genotype 1 infection have implicated single nucleotide polymorphisms (SNPs) within the *IFNL3* (IL28B) gene locus, encoding a lambda interferon, on chromosome 19q13.13 with response to alpha-interferon therapy.^[10, 11, 12]^ Patients with the rs12979860 CC genotype had higher response rates to alpha-interferon.^[13]^ Although the SNP at rs12979860 affects interferon-stimulated gene production as part of the innate immune response, the actual mechanism is unclear.^[14]^ A recent parallel sequencing study suggested that rs4803221 and rs7248668 predicted failure to respond better than did rs12979860.^[15]^ Another recent study showed that rs12979860 is in linkage disequilibrium with a frame-shift variant, ss469415590 [ΔG], which also creates a new gene called *IFNL4* – reduced expression of which may be associated with reduced responsiveness of cells to alpha-interferon.^[16]^ Treatment algorithms incorporating *IFNL3* genotyping are now used in many clinics for the treatment of hepatitis C.^[17]^ Whether the same SNPs affect response to alpha-interferon in other diseases is unclear, but given the role of the innate immune system in the pathogenesis of Behçet’s Syndrome,^[18]^ it is biologically plausible that a similar effect to that seen in hepatitis C with alpha-interferon may be operating in Behçet’s Syndrome. The exploratory analysis in BIO-BEHÇET’S tested this hypothesis.

## Patients and methods

### Objectives

To create an evidence base to underpin effective biologic prescribing for Behçet’s Syndrome, and identify ways to predict and measure response.

### Ethics

Assessed and approved by NRES Committee North West – Liverpool Central (15/NW/0008). Procedures in accordance with ethical standards of the responsible committee on human experimentation and the 1975 Helsinki Declaration, as revised in 1983.

### Study Design

BIO-BEHÇET’S was a pragmatic, standard-of-care, randomized, two-arm, parallel trial comparing biologics after first-line failure. The design and clinical findings have been published previously.^[9]^ Patients with active Behçet’s syndrome with failed response or intolerance to first-line topical steroids or small-molecule immunomodulators were randomized to infliximab (REMICADE; Janssen Biotech Inc) or interferon-α2a (ROFERON; Roche Products) according to relevant protocol in the Behçet’s Syndrome pathway for England.^[19]^ Inclusion and exclusion criteria can be found in the supplementary appendix, along with the dosing schedule (Supplementary Table S1) and information on the trial’s representativeness (Supplementary Table S2). Response was determined as change in Behcet’s Disease Activity Index (BDAI) and significant improvement in organ systems at 12/24-week visits.

### Biomarkers

Based on previous work in hepatitis C – and given the role of the innate immune system in pathogenesis – BIO-BEHÇET’S examined *IFNL3* and *IFNL4* SNPs as biomarkers of response to interferon-α2a and/or infliximab, and the potential for urine metabolomics to act as biomarkers for drug response.

### Genetic Analysis

The genotypic analysis was an exploratory analysis to determine whether any of the SNPs show an effect of efficacy of interferon-α based on primary and secondary outcomes. Test-specific standard operating procedures were written prior to the start of genotyping and strict quality control (QC) measures were adhered to, in order to ensure proper validation of genotype results. If a strong effect was found for a SNP based on one of the primary and secondary outcomes, or as a “trend” over several of the outcomes, the SNP with the highest predictive value was to be tested in approximately 200 other patients (based on power calculations) where DNA is available from our collaborators or in future studies.

Four SNPs within the *IFNL3/4* gene locus were selected owing to a priori knowledge of effects on gene and protein function, or clinical association (Supplementary Table S3).

Genomic DNA was isolated from a 9 mL whole blood sample using an automated Chemagic platform (Perkin Elmer). Genotyping was undertaken and minor allele frequencies counted using off-the-shelf validated allelic discrimination assays (Applied Biosystems). This was carried out by a trained technician with real-time polymerase chain reaction (PCR) utilizing a QuantStudio 6 Fast Real Time PCR System (Applied Biosystems). The following QC thresholds were applied: minor allele frequency (MAF) > 0.05, Hardy Weinberg disequilibrium *P* > 0.0001 and call rate > 95%.

### Metabolomics

Three 1 mL urine samples were collected at baseline, Week 12, 24, and 36. Samples were snap frozen and stored at –80°C before transport to the lab at the Centre for Translational Medicine, The University of Birmingham. After thawing, urine samples were centrifuged at 13,000 ×*g*, prepared using a standard protocol and loaded into a standard 5 mm nuclear magnetic resonance (NMR) tube for spectroscopy. For sample preparation, 450 μL of urine was mixed with 150 μL of 400 mmol/L phosphate buffer at pH 7.0.1D-NOESY presaturation 1H NMR spectra were acquired on a Bruker 600 MHz IvDR NMR system equipped with a z-axis gradient 5 mm triple resonance inverse probe (TXI) probe; 16 steady state scans and a total of 128 transients were acquired per spectrum. All samples were shimmed to achieve a trimethylsilylpropanoic acid (TMSP) linewidth below 1 Hz prior to data acquisition. The spectral width was set to 20 ppm, the interscan relaxation delay was set to 10 s, and a total of 32, 768 complex data points were acquired. All spectra were processed using the MetaboLabPy software ^[20]^ including manual phase correction and data pre-processing. Data pre-processing included excluding regions > 9.8497 ppm, between 6.4522 and 5.6194 ppm and < 0.3168 ppm, segmental alignment of 71 spectral regions, noise filtering, bucketing of 32 datapoints (0.005 ppm), spectral normalization using probabilistic qutionet normalization, variance stabilization using Pareto scaling and finally export into an Excel spreadsheet for statistical data analysis.

One-dimensional 1H spectra were acquired at 300°K using a standard spin-echo pulse sequence with water suppression using excitation sculpting on a Bruker DRX 500 MHz NMR spectrometer equipped with a cryoprobe. Glutamine levels were measured in the urine samples using high-performance ion exchange chromatography, and xanthurenic acid levels were measured using a fluorometric method.

Lists of metabolites providing the greatest discrimination between groups were identified using multivariate analyses and metabolites identified using an NMR database (Human Metabolome Database version 2.5) and Chenomx NMR suite. Strict QC measures were adhered to, ensuring proper validation of genotype results.

### Rationale for Mechanistic Studies

The mechanistic studies were designed to:(a) lead to important developments in the elucidation of the as yet unknown pathophysiological processes underlying Behçet’s Syndrome, (b) clarify the role of two inflammatory pathways involved in a variety of manifestations of the disease and responses (or not) to two distinct biologic drugs that target different inflammatory processes and, (c) identify the potential usefulness of two promising novel biomarkers to facilitate cost-effective targeting of therapy, derived from the greater mechanistic understanding of disease process that (a) and (b) will provide. Assuming the frequency of the CC genotype is 55%, then the power is approximately 75% for detecting a difference in response of 20% (CC genotype 95% versus non-CC genotype 75%, giving an overall response rate of approximately 85%) using a one-sided test and significance level of 0.2. This high significance level is inevitable for a sample size of 45. However, if the overall response rate is 80%, then a difference in response rate of 35% (CC genotype 95% v non-CC genotype 60%) could be detected with 75% power with a 2-sided test and significance level 0.05. To strengthen further the power of the analyses, patient response was also classified on an ordinal scale of “no response”, “poor response”, “good response” according to BDAI score. Results from techniques such as ordinal logistic regression might then be more conclusive. It should be noted that analysis of the mechanistic study is limited to observable differences within treatment groups as opposed to measuring effect of differences between group. This is primarily due to the study sample size but does limit the utility of any conclusions that may be made.

## Results

### Genotyping

Genotypes were obtained for 62 individuals (30 in the infliximab arm, and 32 in interferon-α2a) for all SNPs – with the exception of rs7248668, where a genotype for one individual (interferon-α2a) could not be obtained despite repeated attempts. All SNPs passed the predetermined QC thresholds (Supplementary Table S4). The data suggest that there is high linkage disequilibrium between rs12979860 and rs368234815, and between rs4803221 and rs7248668.

Genotype association with a binary response outcome based on either 20, 50 or 70% response was undertaken using a Pearson’s Chi-squared test for all SNPs in all individuals, plus stratifying for infliximab or interferon-α2a only (Supplementary Table S5). These analyses suggest the only statistically significant associations are for the rs4803221 and rs7248668 SNPs in the interferon-α2a arm, and only when applying the 70% response binary phenotype (*P* = 0.021 and 0.025 respectively); however, after correction for multiple testing (false discovery rate [FDR]), these associations are no longer significant (corrected *P* value [*P*c] > 0.05). Subsequent analysis determined genetic association with four continuous variable outcome measures: BDAI at baseline, 3, and 6 months, and a baseline-adjusted BDAI area under the curve (AUC). This used an ANOVA with Bonferroni correction (Table 1). A notionally statistically significant association was observed for baseline-adjusted BDAI AUC for both rs12979860 and rs368234815 in infliximab patients only (*P* = 0.055); however, this was no longer significant after correction for multiple testing (FDR)(Pc > 0.05).

**Table 1:** IFNL4 SNP associations with BDAI (baseline, 3, and 6 months) and baseline-adjusted BDAI AUC for infliximab, interferon-α2a, and the full cohort

	Infliximab (Mean ± SD)				Interferon-α2a (Mean ± SD)				Overall (Mean ± SD)			
**rs12979860**	**C/C**	**C/T**	**T/T**	**ANOVA *P*-value**	**C/C**	**C/T**	**T/T**	**ANOVA *P*-value**	**C/C**	**C/T**	**T/T**	**ANOVA *P*-value**
BDAI (Baseline)	6.15 ± 2.94	6.67 ± 3.48	7.00	0.877	6.89 ± 2.85	8.07 ± 3.08	8.80 ± 4.71	0.390	6.51 ± 2.88	7.34 ± 3.31	8.50 ± 4.28	0.278
BDAI (3 months)	4.50 ± 3.12	4.58 ± 3.96	7.00 ± 1.41	0.625	6.47 ± 4.00	6.58 ± 4.93	3.67 ± 2.52	0.555	5.52 ± 3.68	5.58 ± 4.49	5.00 ± 2.65	0.956
BDAI (6 months)	4.67 ± 5.92	2.81 ± 1.72	5.50 ± 2.12	0.547	3.94 ± 2.86	5.45 ± 3.78	4.25 ± 5.97	0.559	4.13 ± 4.64	4.13 ± 3.17	4.67 ± 4.76	0.961
Baseline Adj BDAI AUC	-1.21 ± 1.81	-2.00 ± 2.50	2.25 ± 3.89	0.055	-1.31 ± 2.82	-1.27 ± 2.94	-2.25 ± 3.68	0.866	-1.26 ± 2.32	-1.64 ± 2.69	-0.45 ± 4.08	0.639
**rs368234815**	**TT/TT**	**TT/G**	**G/G**	**ANOVA *P*-value**	**TT/TT**	**TT/G**	**G/G**	**ANOVA *P*-value**	**TT/TT**	**TT/G**	**G/G**	**ANOVA *P*-value**
BDAI (Baseline)	6.15 ± 2.94	6.67 ± 3.48	7.00	0.877	6.83 ± 2.92	8.07 ± 2.96	8.80 ± 4.71	0.367	6.47 ± 2.91	7.37 ± 3.25	8.50 ± 4.28	0.254
BDAI (3 months)	4.50 ± 3.12	4.58 ± 3.96	7.00 ± 1.41	0.625	5.93 ± 3.45	7.23 ± 5.26	3.667 ± 2.52	0.401	5.22 ± 3.32	5.96 ± 4.78	5.00 ± 2.65	0.748
BDAI (6 months)	4.67 ± 5.92	2.81 ± 1.72	5.50 ± 2.12	0.547	3.94 ± 2.95	5.33 ± 3.63	4.25 ± 5.97	0.601	4.32 ± 4.71	4.13 ± 3.09	4.67 ± 4.76	0.959
Baseline Adj BDAI AUC	-1.21 ± 1.81	-2.00 ± 2.50	2.25 ± 3.89	0.055	-1.55 ± 2.72	-0.96 ± 3.01	-2.25 ± 3.68	0.754	-1.37 ± 2.26	-1.46 ± 2.76	-0.45 ± 4.08	0.732
**rs4803221**	**C/C**	**C/G**	**G/G**	**ANOVA *P*-value**	**C/C**	**C/G**	**G/G**	**ANOVA *P*-value**	**C/C**	**C/G**	**G/G**	**ANOVA *P*-value**
BDAI (Baseline)	6.12 ± 2.81	7.11 ± 4.08	7.00	0.706	7.19 ± 3.22	8.20 ± 3.16	9.50 ± 3.54	0.489	6.65 ± 3.04	7.68 ± 3.56	8.67 ± 2.89	0.319
BDAI (3 months)	4.15 ± 2.89	5.78 ± 4.41	6.00	0.468	6.27 ± 4.67	6.44 ± 3.43	4.00	0.868	5.26 ± 4.02	6.11 ± 3.85	5.00 ± 1.41	0.736
BDAI (6 months)	4.45 ± 5.39	2.63 ± 1.69	7.00	0.537	4.05 ± 3.20	6.50 ± 4.17	1.50 ± 2.12	0.117	4.25 ± 4.38	4.56 ± 3.67	3.33 ± 3.51	0.892
Baseline Adj BDAI AUC	-1.26 ± 1.66	-1.38 ± 3.88	-0.50	0.943	-1.25 ± 2.75	-1.16 ± 2.92	-6.25	0.226	-1.26 ± 2.24	-1.27 ± 3.32	-3.38 ± 4.07	0.530
**rs7248668**	**G/G**	**G/A**	**A/A**	**ANOVA p-value**	**G/G**	**G/A**	**A/A**	**ANOVA p-value**	**G/G**	**G/A**	**A/A**	**ANOVA p-value**
BDAI (Baseline)	6.12 ± 2.81	7.25 ± 4.33	7.00	0.672	7.19 ± 3.23	8.33 ± 3.32	9.50 ± 3.54	0.470	6.65 ± 3.04	7.82 ± 3.75	8.67 ± 2.89	0.290
BDAI (3 months)	4.15 ± 2.89	5.78 ± 4.41	6.00	0.468	6.27 ± 4.67	6.50 ± 3.66	4.00	0.869	5.26 ± 4.02	6.12 ± 3.97	5.00 ± 1.41	0.745
BDAI (6 months)	4.45 ± 5.39	2.63 ± 1.69	7.00	0.537	4.05 ± 3.20	5.57 ± 3.51	1.50 ± 2.12	0.276	4.25 ± 4.38	4.00 ± 3.00	3.33 ± 3.51	0.920
Baseline Adj BDAI AUC	-1.26 ± 1.66	-1.38 ± 3.88	-0.50	0.943	-1.25 ± 2.75	-1.46 ± 3.02	-6.25	0.238	-1.26 ± 2.44	-1.42 ± 3.38	-3.38 ± 4.07	0.523

Data represent mean ± standard deviation. Statistical significance determined by one-way ANOVA.

### Metabolomics

**Figure 1:**
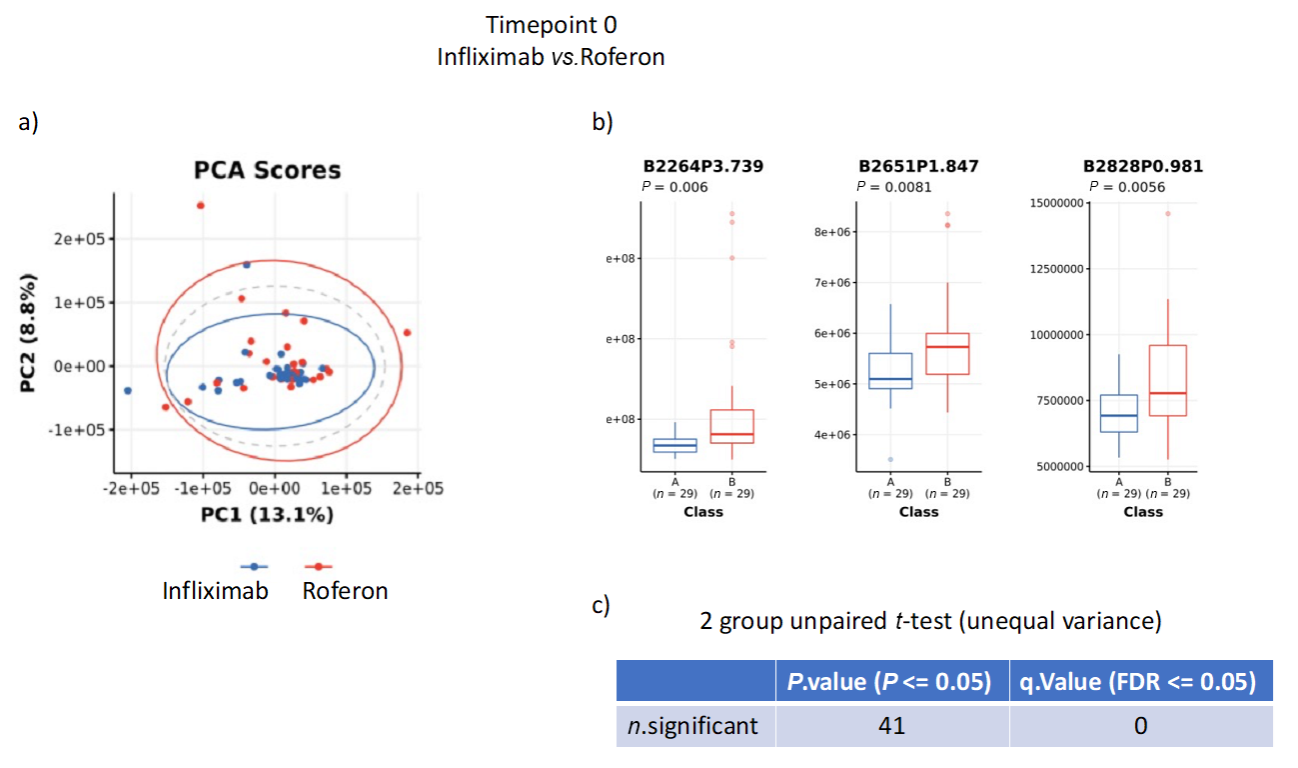
a) Principal contents analysis (PCA) of baseline urine samples prior to randomized drug treatment; b) specific bins significantly different between groups; c) unpaired t-test for multiple comparisons.

Initial results showed no significant metabolite differences at baseline between patients allocated to infliximab or interferon-α2a (Figure 1a). NMR data were analyzed as bins containing multiple metabolites. Three bins showed significant differences between the two treatment groups (Figure 1b); however, none remained significant when corrected for multiple comparison of all bins (Figure 1c).

**Figure 2:**
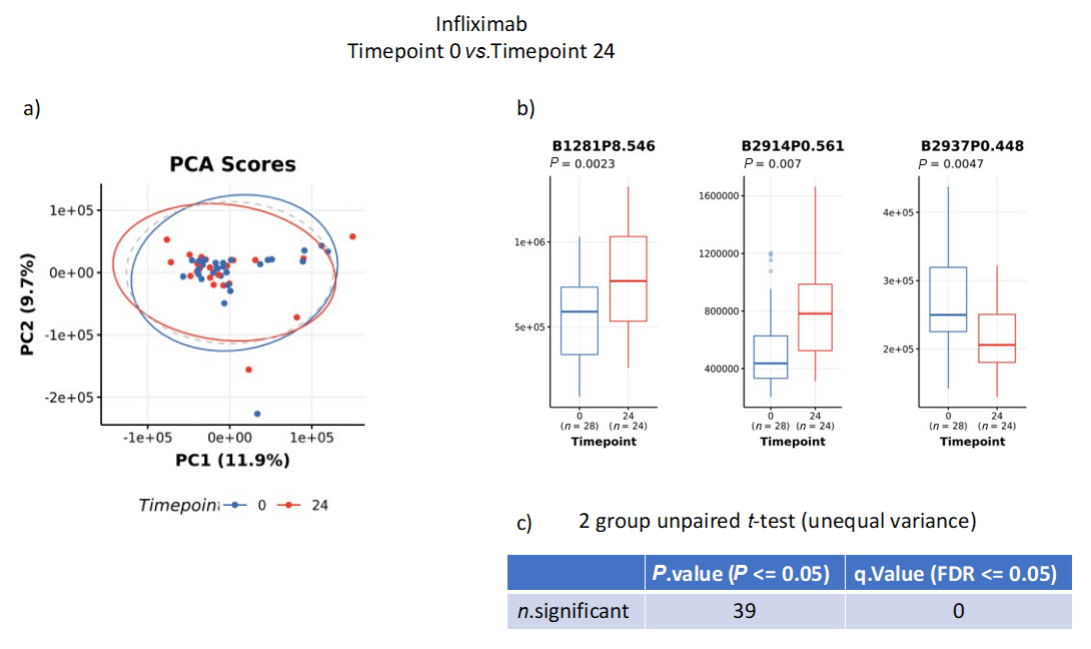
Analysis of nuclear magnetic resonance (NMR) analysis of urine samples from patients on infliximab at baseline versus 24 weeks. a) principal contents analysis (PCA); b) significant bins; c) unpaired t-test for multiple comparisons.

**Figure 3:**
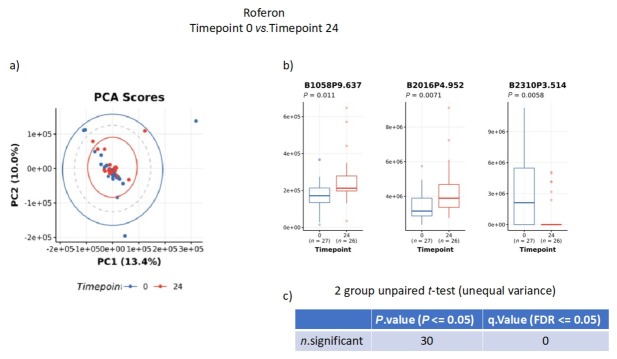
Analysis of nuclear magnetic resonance (NMR) analysis of urine samples from patients on interferon-α2a at baseline versus 24 weeks. a) principal contents analysis (PCA); b) significant bins; c) unpaired t-test for multiple comparisons.

To test whether drug treatments altered metabolite profiles, baseline samples from patients on infliximab (Figure 2) or interferon-α2a (Figure 3) were compared to samples from the same patient taken at Week 24. Principal component analysis (PCA) results showed no major difference in metabolite profiles between the two samples. Specific bins did show significance, though this was lost when correction for multiple comparisons was made.

**Figure 4:**
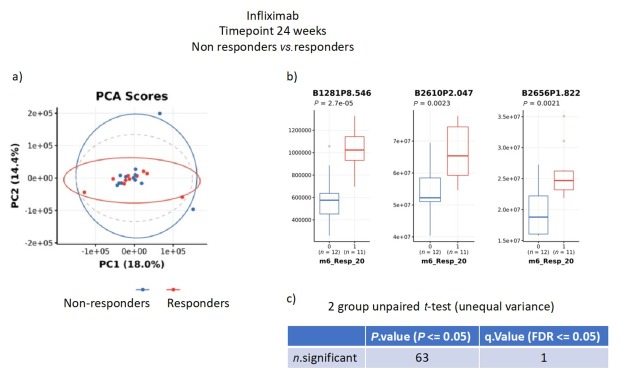
nuclear magnetic resonance (NMR) metabolite analysis of urine samples from responders to Infliximab compared to non-responders. a) principal contents analysis (PCA); b) significantly different specific bins; c) unpaired t-tests for multiple comparisons.

To determine whether metabolite profiles were associated with response to the individual drugs, urine samples at Week 24 from responders and non-responders were compared. Samples from patients on infliximab showed similar clustering by PCA analysis (Figure 4). Specific bins once again showed significant differences, with one bin (B1281P8.546) remaining significant after correction for multiple comparisons.

**Figure 5:**
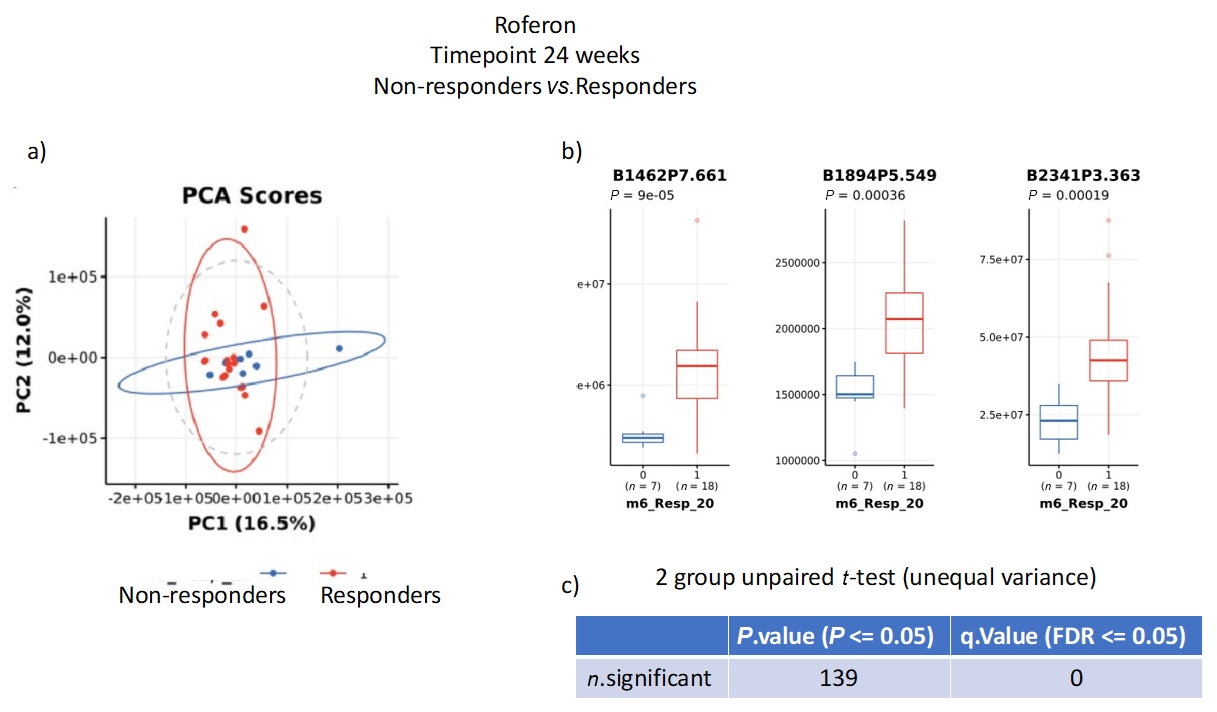
nuclear magnetic resonance (NMR) metabolite analysis of urine samples from responders to Interferon-α2a compared to non-responders. a) principal contents analysis (PCA); b) significantly different specific bins; c) unpaired t-tests for multiple comparisons.

For responders versus non-responders to interferon-α2a, there was weak separation between the groups by PCA analysis, particularly clustering of responder samples (Figure 5). This pattern may have been due to specific bins which were significantly different between the groups, although such significance was lost when corrected for multiple comparison.

## Discussion

BIO-BEHÇET’S included a mechanistic component to address the potential to predict response to infliximab or interferon-α2a treatment, which each possess distinct modes of action. Genetics and urine metabolomics were used to further improve cost effectiveness and precision. Genotyping for four SNPs in the *IFNL3* (IL28B) gene locus was undertaken based on literature supporting their role in predicting viral clearance for hepatitis C infection and NMR-based urine metabolomics, which have shown promise in predicting response in rheumatoid arthritis.^[21]^

Genetic data are suggestive of an association between patient outcome and carriage of either rs4803221 or rs7248668 variants in the *IFNL3* (IL-28B) gene locus – but for the interferon-α2a arm only. This observation is aligned with previous findings, which demonstrate significant association between these two SNPs and interferon-α2a treatment outcomes in hepatitis C.^[12]^ This may be indicative of the potential to predict patient outcomes to interferon-α2a according to genotype in this patient population. However, given the relatively small sample size, and the fact that the statistical significance of the association is negated by correction for multiple testing, the results must be treated with caution. Larger, adequately powered genetic studies are required to verify this finding in the context of Behçet’s Syndrome.

BIO-BEHÇET’S is the first prospective head-to-head study to utilize metabolomics to examine the potential for differential effects of two biologics in the treatment of Behçet’s Syndrome. Metabolomic analysis showed no significant differences between the patients at baseline before randomization, confirming that there were no major confounding factors that may have influenced response to a particular treatment. Analysis between individual patient urine metabolite profiles at baseline compared to 24 weeks indicated no major differences, suggesting the drugs were not inducing wide-ranging systemic changes to metabolic processes, rather that any effects would be due to specific metabolic changes to each drug’s target pathway. This was supported by comparison of 24-week urine samples from responders and non-responders to the same drug. For infliximab, one bin remained significant after multiple correction, though PCA clustering was weaker for patients on interferon-α2a.

Whilst of interest, it should be noted that comparison between responders and non-responders split each group, leading to a smaller number of samples for analysis. However, the results for infliximab seem to indicate that within one of the bins we have detected potential marker (s) of a metabolic response to treatment, which is worthy of further study to identify the individual metabolites and associated metabolic pathways responsible. The signal was weaker for interferon-α2a, and may not necessarily be due to the same metabolites and pathways. The specific bins that vary between each comparison will therefore be further analysed to determine the specific metabolites responsible for this difference, which may identify the particular pathways being influenced by each treatment.

A limitation of this study was using only urine and NMR analysis of metabolites. Analysis of urine samples from patients with Behçet’s Syndrome and healthy controls using mass spectroscopy identified a biomarker panel of 10 metabolites which showed clear discrimination of the groups.^[22]^ But changes in serum lipid markers have been described previously in patients with Behçet’s Syndrome and healthy controls using mass spectrometry analysis.^[23,24]^ Amino acids including glutamate and valine have also been identified as potential biomarkers in synovial fluid from patients with Behçet’s Syndrome with arthritis compared to patients with sero-negative arthritis.^[25]^ Furthermore, genetic variability, acquired or innate antibodies, receptor dysregulation, or antibodies stimulated by treatment may modulate clinical responsiveness to interferon-α2a. Increased frequencies of anti-interferon (IFN)-α and various autoantibodies associated with interferon-α2a treatment have been suggested to be associated with a better clinical response.^[26]^ These aspects were not examined in this study.

## Conclusion

BIO-BEHÇET’S advances existing knowledge by comparing two drug therapies in patients with Behçet’s Syndrome. It also supports the randomization process of patients and helps direct future research towards the direct effect of each drug-limiting process, rather than altering them in a patient’s response to treatment.

The genetic data suggest the possibility of an association between response to treatment and carriage of either rs4803221 or rs7248668 variants in the *IFNL3* (IL-28B) gene locus for the interferon-treated arm in line with association between these two SNPs and previously observed interferon-α2a treatment outcomes in hepatitis C.

As this trial was powered for the primary outcome of clinical efficacy, these exploratory biomarker results must be treated with caution due to small numbers in responder subgroups. But there were no baseline differences in metabolomic analysis between the patients before randomization, indicating no major confounding factors that may have influenced response to a particular treatment. Comparison of 24-week urine samples from responders and non-responders to the same drug using principal component analysis revealed for infliximab one specific bin of metabolites that remained significantly different comparing responders to non-responders. This effect was weaker for interferon-α2a. Further work will characterize the appropriate metabolite (s) from existing samples to inform future prospective trials, which may be designed as prospective cohort studies, or real-world studies, to allow this to be explored in more detail and with more patients clinically.

## Supplementary Information

*Supplementary materials are only available at the official site of the journal (www.rir-journal.com*).
